# The antiviral effect of metformin on zika and dengue virus infection

**DOI:** 10.1038/s41598-021-87707-9

**Published:** 2021-04-22

**Authors:** Carlos Noe Farfan-Morales, Carlos Daniel Cordero-Rivera, Juan Fidel Osuna-Ramos, Irma Eloisa Monroy-Muñoz, Luis Adrián De Jesús-González, José Esteban Muñoz-Medina, Arianna M. Hurtado-Monzón, José Manuel Reyes-Ruiz, Rosa María del Ángel

**Affiliations:** 1 Department of Infectomics and Molecular Pathogenesis, Center for Research and Advanced Studies (CINVESTAV-IPN), Mexico City, Mexico; 2 Department of Genetics and Human Genomics, National Institute of Perinatology “Isidro Espinosa de Los Reyes”, Mexico City, Mexico; 3 Central Laboratory of Epidemiology, National Medical Center la Raza, Mexican Institute of Social Security, Mexico City, Mexico

**Keywords:** Drug discovery, Medical research, Pathogenesis

## Abstract

The Dengue (DENV) and zika (ZIKV) virus infections are currently a public health concern. At present, there is no treatment or a safe and effective vaccine for these viruses. Hence, the development of new strategies as host-directed therapy is required. In this sense, Metformin (MET), an FDA-approved drug used for the treatment of type 2 diabetes, has shown an anti-DENV effect in vitro by activating AMPK and reducing HMGCR activity. In this study, MET treatment was evaluated during in vitro and in vivo ZIKV infection and compared to MET treatment during DENV infection. Our results demonstrated that MET has a broad in vitro antiviral spectrum. MET inhibited ZIKV infection in different cell lines, but it was most effective in inhibiting DENV and yellow fever virus (YFV) infection in Huh-7 cells. However, the drug failed to protect against ZIKV infection when AG129 immunodeficient mice were used as in vivo model. Interestingly, MET increased DENV-infected male mice's survival time, reducing the severe signs of the disease. Together, these findings indicate that, although MET was an effective antiviral agent to inhibit in vitro and in vivo DENV infection, it could only inhibit in vitro ZIKV infection.

## Introduction

The reappearance of different arboviruses around the world has generated a worldwide health alert. Among arbovirus, the genus flavivirus comprises more than 50 different viral species, and some of them are emerging in new geographic areas, such as dengue (DENV) and Zika virus (ZIKV). Both viruses can induce different clinical conditions ranging from fever to severe disease complications and even death^1,2^.

The ZIKV has spread exponentially across the Americas, causing one of the most critical outbreaks in this century with terrible consequences in human health due to its association with the post-zika fetal and Guillain-Barré syndrome^3^. On the other hand, the infection caused by the four DENV serotypes continues to be a health problem worldwide due to its high incidence, with outbreaks of increasing frequency and magnitude in the last 50 years in the Americas^4^. DENV infection is estimated to cause 390 million infections and 20,000 deaths per year^1,5^.

Currently, there is no vaccine or specific therapeutic drug against ZIKV, and the scientific community's efforts to develop a vaccine or medication for the different flaviviruses infections continue^6^. Besides, the use of the FDA-approved dengue vaccine (Dengvaxia CYD-TDV) has been restricted to people living in endemic areas, ranging from 9 to 45 years old, who have had at least one previously documented DENV infection^7^. Therefore, the search for new strategies to control diseases caused by these viruses is essential.

The flaviviruses are enveloped viruses with icosahedral nucleocapsids of 40–60 nm diameter and whose genetic material consists of a single chain of positive polarity RNA of approximately 10,000 to 11,000 bases^8^. The viral lipid membrane is acquired from the endoplasmic reticulum during its replicative cycle; therefore, these viruses' viral cycle is strongly linked to the metabolism of host cell lipids^9,10^. In vitro assays have shown the importance of lipids, such as cholesterol, during viral replication^11,12^. Moreover, flavivirus infections are inhibited by drugs that reduce the synthesis and/or import of cholesterol^13,14^.

Among FDA-approved drugs, lipid-lowering drugs are an alternative for the treatment of flavivirus infections. For instance, Lovastatin (LOV) has shown an antiviral effect in vitro^15^ and in vivo^16^*;* therefore, it has been postulated as a therapeutic candidate due to its safe use and low cost. However, no evidence of a beneficial effect on any clinical manifestations of DENV or viremia has been found in adult patients treated with LOV^17^.

Metformin (MET), a drug widely used to treat type II diabetes due to its excellent safety profile, is another candidate for the treatment of DENV. In addition to its hypoglycemic effect^18,19^, MET can reduce lipid synthesis by activating AMP-activated protein kinase (AMPK), the master regulator of cellular metabolism^20,21^.

Our group described for the first time that DENV infections are sensitive to in vitro MET treatments that activate the AMPK, which can reduce cell cholesterol synthesis by decreasing the activity of 3-hydroxy-3-methyl-glutaryl-coenzyme A reductase (HMGCR)^14^.

Subsequently, a retrospective cohort study in diabetic adults reported the therapeutic potential of MET to reduce severe forms of dengue disease^22^. Also, MET currently being tested as adjunctive therapy for dengue in overweight and obese patients^23^. Therefore, this article has focused on studying the potential of MET to inhibit in vitro and in vivo ZIKV infection, comparing it with its anti-DENV effect.

## Results

### MET reduces the ZIKV infection in different cell lines

**Figure 1:**
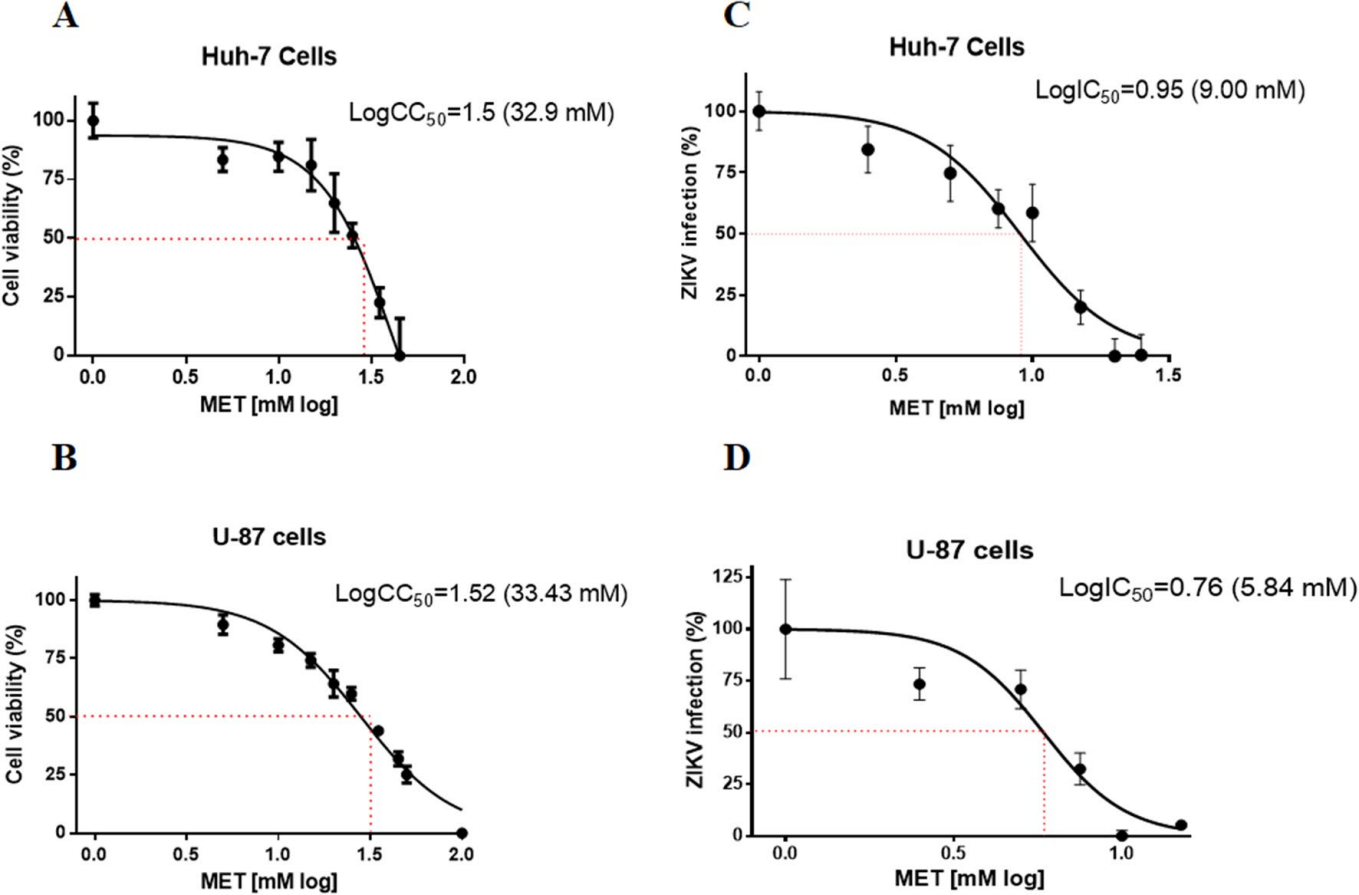
Cytotoxicity (CC_50_), inhibition concentration (IC_50_), and selectivity index (SI) of MET in Huh-7 and U-87 cell lines infected with ZIKV. The CC_50_ on Huh-7 (**A**) and U-87 (**B**) cells were calculated with the vehicle (H_2_O) or increasing concentrations of MET (1, 2.5, 5, 7.5, 10, 15, 20, and 25 mM). The cell viability was evaluated by MTT assay at 24 h, and a linear regression analysis estimated the CC_50_. The IC_50_ was calculated for MET in ZIKV-infected Huh-7 (**C**) and U-87 (**D**) cells. Both cell lines were treated with MET (1, 2.5, 5, 7.5, 10, 15, 20, and 25 mM) or vehicle (H2O) for 24 h. The reduction of infection by MET was evaluated by flow cytometry, and the IC_50_ was estimated by linear regression analysis. The results were plotted with Graph Pad Prism software version 6.0.

The human hepatocarcinoma cells (Huh-7) and glioblastoma cells (U-87) were used to determine the effect of MET on ZIKV infection. For this purpose, the selectivity index (SI) of MET based on the CC_50_/IC_50_ ratio was calculated in both cells. The MET cytotoxicity was similar in both cell lines; Huh-7 cells showed a CC_50_ of 32.9 mM and 33.43 mM in U-87 cells (Fig. 1A,B). Then, the IC_50_ of MET was calculated in both cell lines. First, we determined the efficacy of infection in both lines, finding no statistically significant differences (Figure S1B); this made it possible to compare its effectiveness between both cell types. MET inhibited ZIKV infection in both cell lines in a dose-dependent manner with an IC_50_ of 9.0 mM (95% CI = 7.36 to 11.25 mM) and 5.84 (95% CI = 4.96 to 6.87 mM), respectively (Fig. 1C,D). Therefore, the calculated values of the SI for each cell type were 4.68 and 3.65 for Huh-7 and U-87 cells, respectively (Table 1).

**Table 1:** Selectivity index of MET.

Flavivirus	Cells	CC_50_	IC_50_	SI (CC_50_/IC_50_)
ZIKV	U-87	33.43	5.84	5.72
ZIKV	Huh-7	32.9	9	3.65
DENV	Huh-7	32.9	3.82	8.61
YVF	Huh-7	32.9	5.36	6.13

Cytotoxicity (CC_50_), anti-flavivirus activity (IC_50_), selectivity index (SI).

### MET treatment reduces the viral yield and viral protein synthesis in ZIKV-infected Huh-7 and U-87 cells

**Figure 2:**
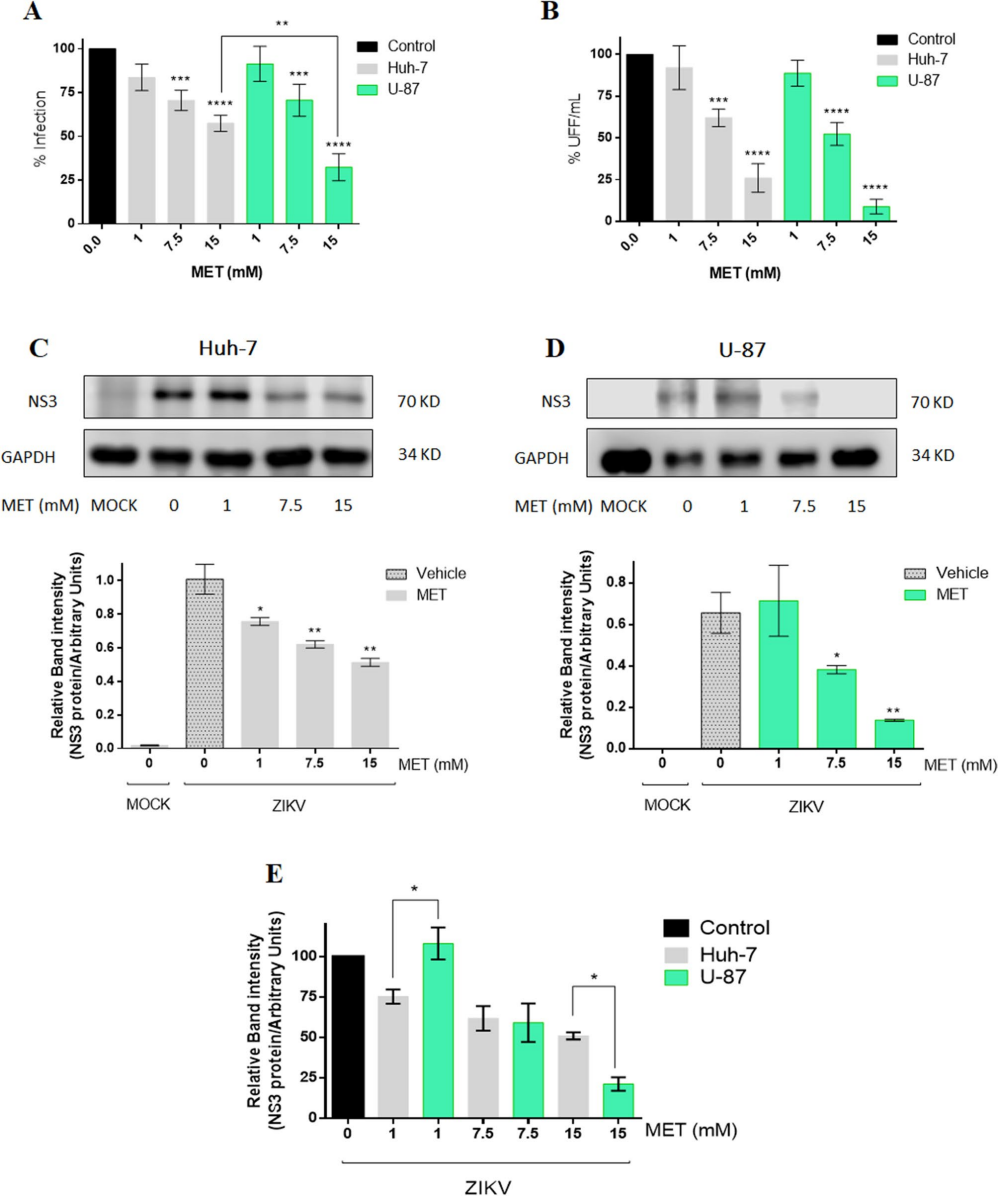
Met reduces viral yield and viral protein synthesis during the ZIKV infection in Huh-7 and U-87 cells. The Huh-7 and U-87 infected cells were treated with Met (1,7.5,15 mM) for 24 h. (**A**) Infected cells analyzed by flow cytometry. (**B**) Viral titers of the collected supernatants analyzed by foci forming units (FFU) assay. The NS3-ZIKV protein was analyzed by western blot in (**C**) Huh-7 cells, and (**D**) U-87 cells; the densitometric analysis is shown below. (**E**) Statistical analysis of densitometry between both cell lines. The results are presented in percentages considering as 100% the infected cells untreated (Control). The graphs represent the means ± SD of n = 3 independent experiments realized by duplicated. *p = 0.0332, **p = 0.0021, ***p = 0.0002 ****p < 0.0001.

The antiviral effect of MET on ZIKV infection was evaluated using three concentrations: 1 mM (low), 7.5 (medium), and 15 mM (high). The concentrations were chosen based on MET toxicity in Huh-7 and U-87 cells (Figure S1A). At the end of the treatments, supernatants were collected to determine viral yield by foci forming units (FFU) assay, while the percentage of infection was analyzed by flow cytometry. Treatment with MET reduced the percentage of infection (− 29.43% at 7.5 mM, and − 42.59% at 15 mM) and viral yield (− 38% at 7.5 mM, and − 74.00% at 15 mM) in a dose-dependent manner in Huh-7 cells (Fig. 2A,B). Besides, the viral protein synthesis was evaluated by quantifying NS3 protein levels by western blot assay. The NS3 protein levels (Fig. 2C) in Huh-7 treated cells were reduced in all concentrations (− 24%, − 38.15%, and − 48.95).

Similarly, in U-87 cells (Fig. 2A,B,D), MET reduced the percentage of infected cells (− 30.36% at 7.5 mM, and − 67.59% at 15 mM), viral yield (− 47.69% at 7.5 mM, and − 91.13% at 15 mM), and NS3 viral protein levels (− 40.80% at 7.5 mM, and − 78.70% at 15 mM). Statistical analysis between both cell types showed a greater antiviral effect in U87 cells with significant differences in the percentage of infection (p = 0.0054) and viral protein levels (p = 0.0220) between both cell lines at the highest concentration (Fig. 2A,E).

### MET treatment inhibits DENV more effectively than ZIKV infection in Huh-7 cells

**Figure 3:**
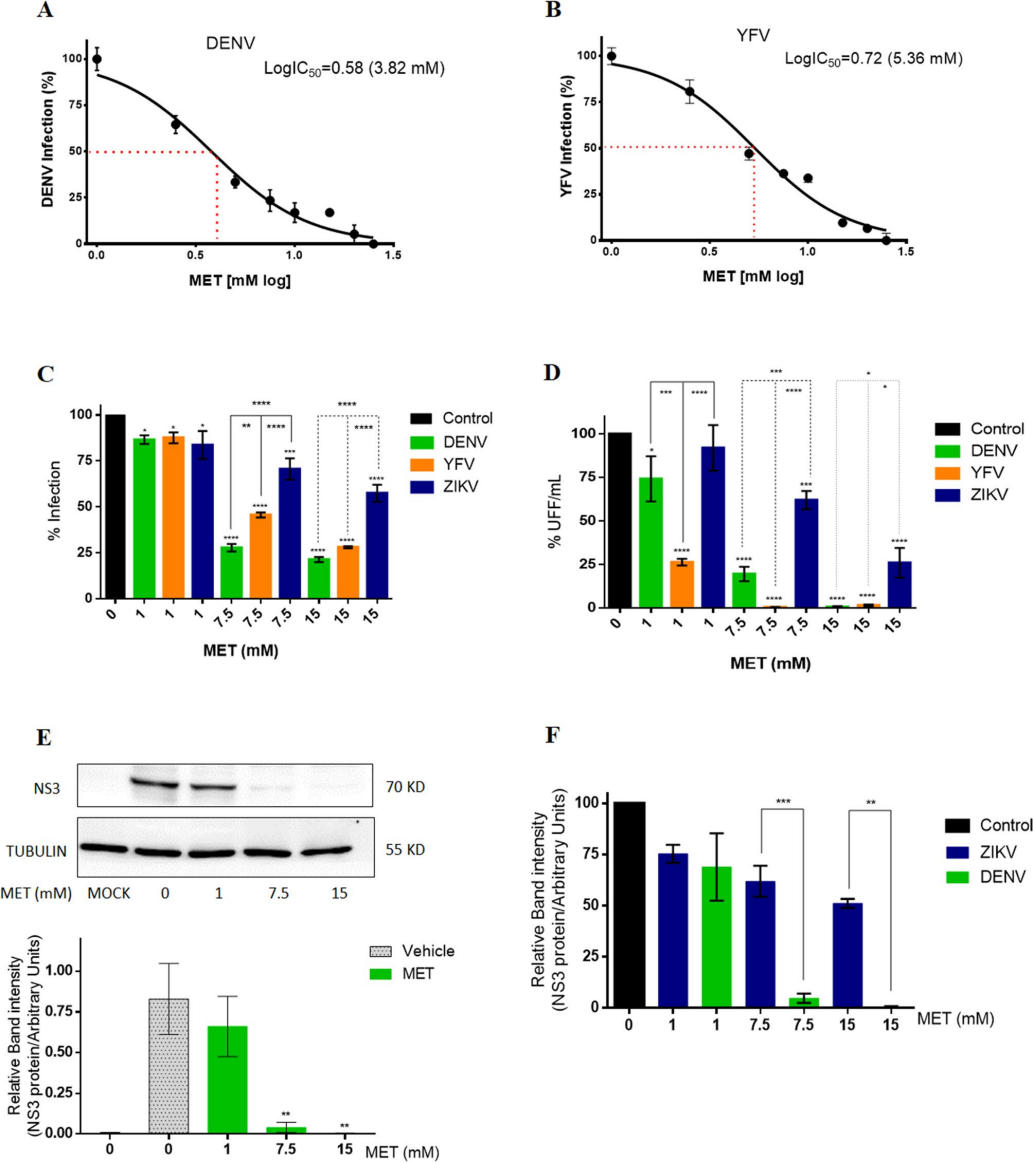
Effect of MET in DENV, YFV, and ZIKV infection. The CC_50_ of MET for (**A**) DENV and (**B**) YFV was calculated in Huh-7 cells. The reduction of viral infection by MET was evaluated by flow cytometry, and the IC_50_ was estimated by linear regression analysis. (**C**) Percentage of infection and (**D**) viral yield of DENV, YFV, and ZIKV in the presence of different concentrations of MET (1,7.5,15 mM) for 24 h. The cells were analyzed by flow cytometry and the viral titers of the collected supernatants analyzed by focus forming units (FFU) assay. (**E**) The DENV NS3 protein levels were analyzed by western blot in Huh-7 cells after treatment with different concentrations of MET. The densitometric analysis is shown. (**F**) Densitometric analysis comparing the DENV and ZIKV NS3 protein levels after treatment with different concentrations of MET. The results are presented in percentages considering as 100% the untreated infected cells (Control). The results were plotted with Graph Pad Prism software version 6.0. The graphs represent the means ± SD of n = 3 independent experiments realized by duplicated. *p = 0.0332, **p = 0.0021, ***p = 0.0002 ****p < 0.0001.

The IC_50_ and SI of MET were calculated for DENV and YFV infection to determine the efficacy of MET to inhibit other flaviviruses. The infection efficacy of each virus is shown in Figure S1B. MET inhibited the infection of both flaviviruses in a dose-dependent manner, showing 3.82 (95% CI = 3.350 to 4.362) and 5.36 (95% CI = 4.892 to 5.893) of IC_50_ values for DENV and YFV, respectively (Fig. 3A,B) in Huh-7 cells. The calculated SI value for DENV and YFV was 8.61 and 6.13, respectively (Table 1). Subsequently, using the previously described concentrations (1, 7.5, and 15 mM), the effect of MET on the flaviviruses infection was compared (Fig. 3C,D). MET treatment reduced the percentage of infection (− 13.46%, − 72.24%, and − 78.65%) and viral yield of DENV (− 25.84%, − 80.42%, and − 99.38%), in a dose-dependent manner. Likewise, the treatment reduced the percentage of infection (− 12.47%, − 54.41%, and − 71.98%) and the viral yield of YFV (− 25.84%, − 80.42%, and − 99.38%). The statistical analysis showed that the antiviral activity of MET was higher against DENV and YFV compared to ZIKV (Fig. 3C,D). In this sense, MET inhibited more the percentage of DENV infection than the percentage of YFV (at 7.5 mM) and ZIKV (at 7.5 and 15 mM) infection. Interestingly, MET decreased in more extent the viral yield of YFV than that of DENV (at 1 mM) and ZIKV (at 1, 7.5, and 15 mM). The effectiveness of MET to inhibit ZIKV and DENV infection was corroborated by WB assay (Fig. 3E,F). MET treatment (at 7.5 and 15 mM) significantly reduced the NS3 protein levels of DENV infected cells compared to ZIKV-infected cells.

### MET treatment inhibits the flavivirus replication complex formation in Huh-7 cells

**Figure 4:**
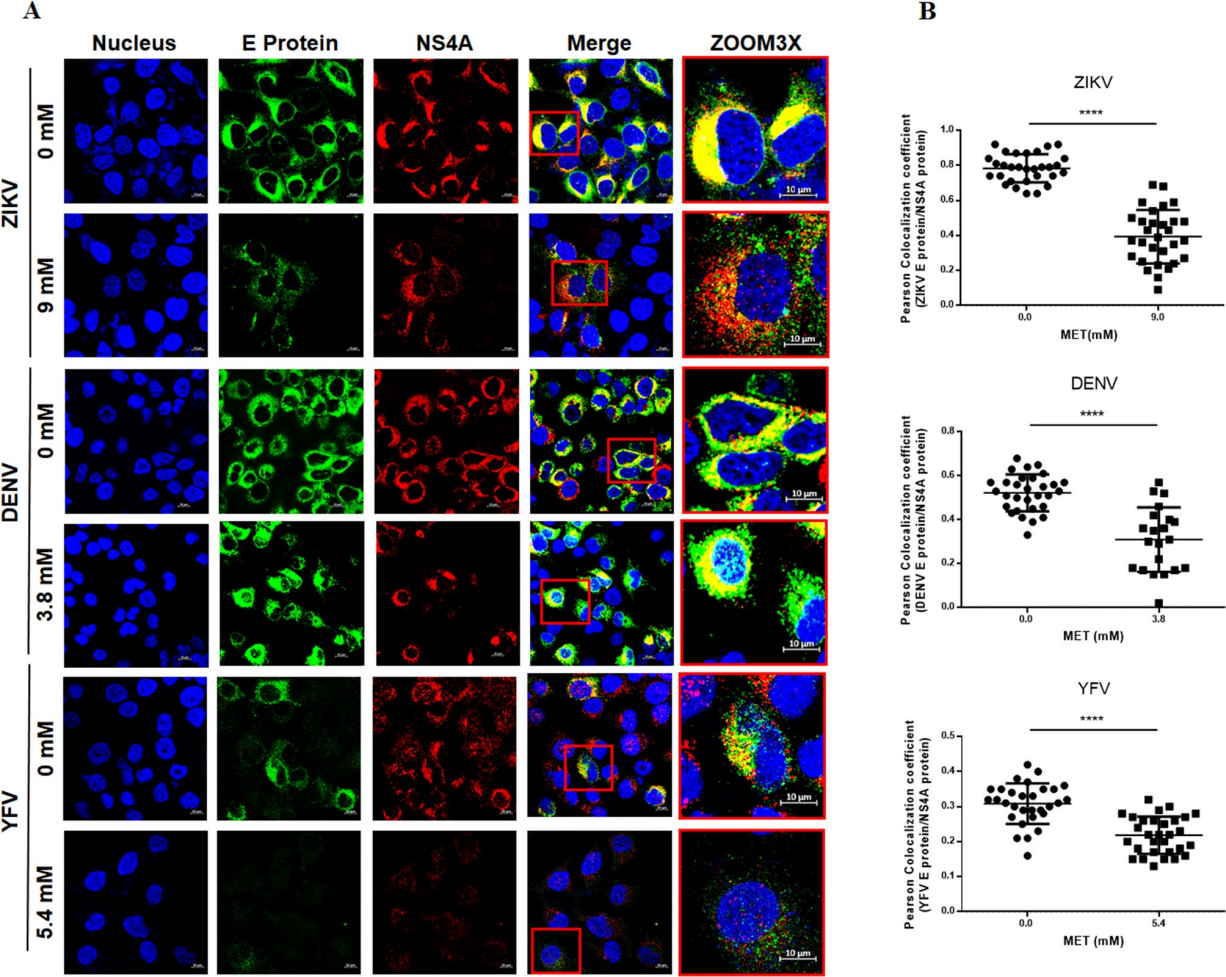
MET inhibits replicative complex formation during flavivirus infection. (**A**) The distribution of the E (green) and NS4A (red) viral proteins present at Flaviviruses-replication complexes was evaluated by confocal microscopy in Huh7 cells treated with MET and infected (MOI 5) with ZIKV, DENV, and YFV at 24 h. Nuclei were stained with Hoechst (blue). Red boxes are depicting the zoom area. Scale bar 10 μm. (**B**) Semi-quantitative analysis of the Pearson correlation between treated and untreated cells; in all infected cells, NS4A colocalized with E protein, while in the treated cells, they presented delocalization. The graphics indicate E/NS4A colocalization values for the region of interest (ROI) of 30 infected cells from three independent images expressed as mean ± SD. ****p < 0.0001.

**Figure 5:**
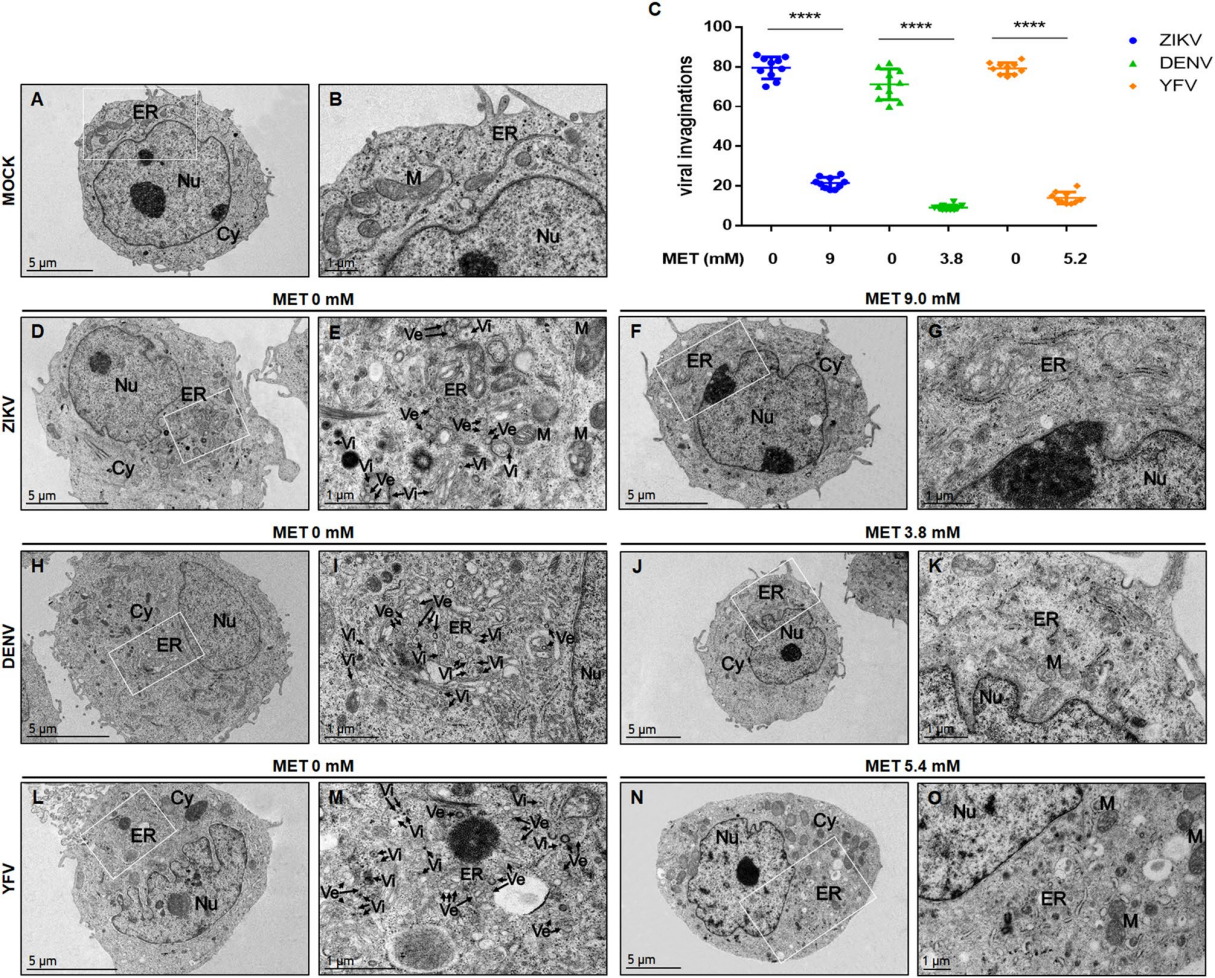
MET reduces the formation of structures associated with the replication complexes of flaviviruses in Huh-7 cells. Transmission electron microscopy of Huh-7 cells infected with mock (**A**,**B**), ZIKV (**D**,**E**), DENV (**H**,**I**), YFV (**L**,**M**) and treated with MET (**F**,**G**,**J**,**K**,**N**,**O**). *Nu* nucleus, *Cy* cytoplasm, *ER* endoplasmic reticulum, *M* mitochondria, *Ve* virus-induced vesicles, *Vi* virus-like particles. (**C**) The graph represents the Ve count of treated cells compared to untreated cells, expressed as the mean ± SD. n = 10 cells per group. **** p < 0.0001.

In order to confirm the antiviral effect of MET in DENV, ZIKV and YFV infection, Huh-7 cells were infected with each virus separately and treated with MET using the IC_50._ Subsequently, replicative complex formation was evaluated by confocal microscopy, as previously reported by Soto-Acosta et al.^14^. The presence of replicative complexes was evidenced by the colocalization of the two viral proteins, E (green) and NS4A (red). MET treatment reduced the fluorescence of both viral proteins (E and NS4A) in the perinuclear region of flavivirus-infected Huh-7 cells (Fig. 4A). The semi-quantitative analysis of the Pearson correlation coefficient showed statistically significant differences between treated and untreated cells (p < 0.0001) (Fig. 4B), suggesting that MET inhibits the replicative complex formation of the three analyzed flaviviruses. To further confirm these results, the ultrastructural of Huh-7 cells infected with ZIKV, DENV, and YFV and untreated or treated with MET was evaluated by transmission electron microscopy (TEM) (Fig. 5A). Untreated cells lost the endoplasmic reticulum (ER) structure due to the formation of replicative complexes characterized by numerous invaginations and membranous structures (Fig. 5D,E,H,I,L,M). In contrast, MET treatment reduced the virus-induced vesicles^24^ (Ve) in ZIKV, DENV, and YFV-infected Huh-7 cells (Fig. 5F,G,J,K,N,O). Besides, no virus-like particles (Vi) were detected during MET treatments. The semi-quantitative analysis of virus-induced vesicles also showed less replicative complexes in MET-treated cells than in untreated cells (p < 0.0001), as shown in Fig. 5C.

### MET protects AG129 mice against DENV but not ZIKV infection

The AG129 mouse, usually used as an animal model for DENV and ZIKV infection, was employed to determine the antiviral effect of MET. Mice were infected intraperitoneally with 2 × 10^7^ FFU of ZIKV or 4 × 10^6^ FFU of DENV per mouse. The treated group received 50 mg/kg/day of MET for ten days, starting on the fourth day post-infection.

**Figure 6:**
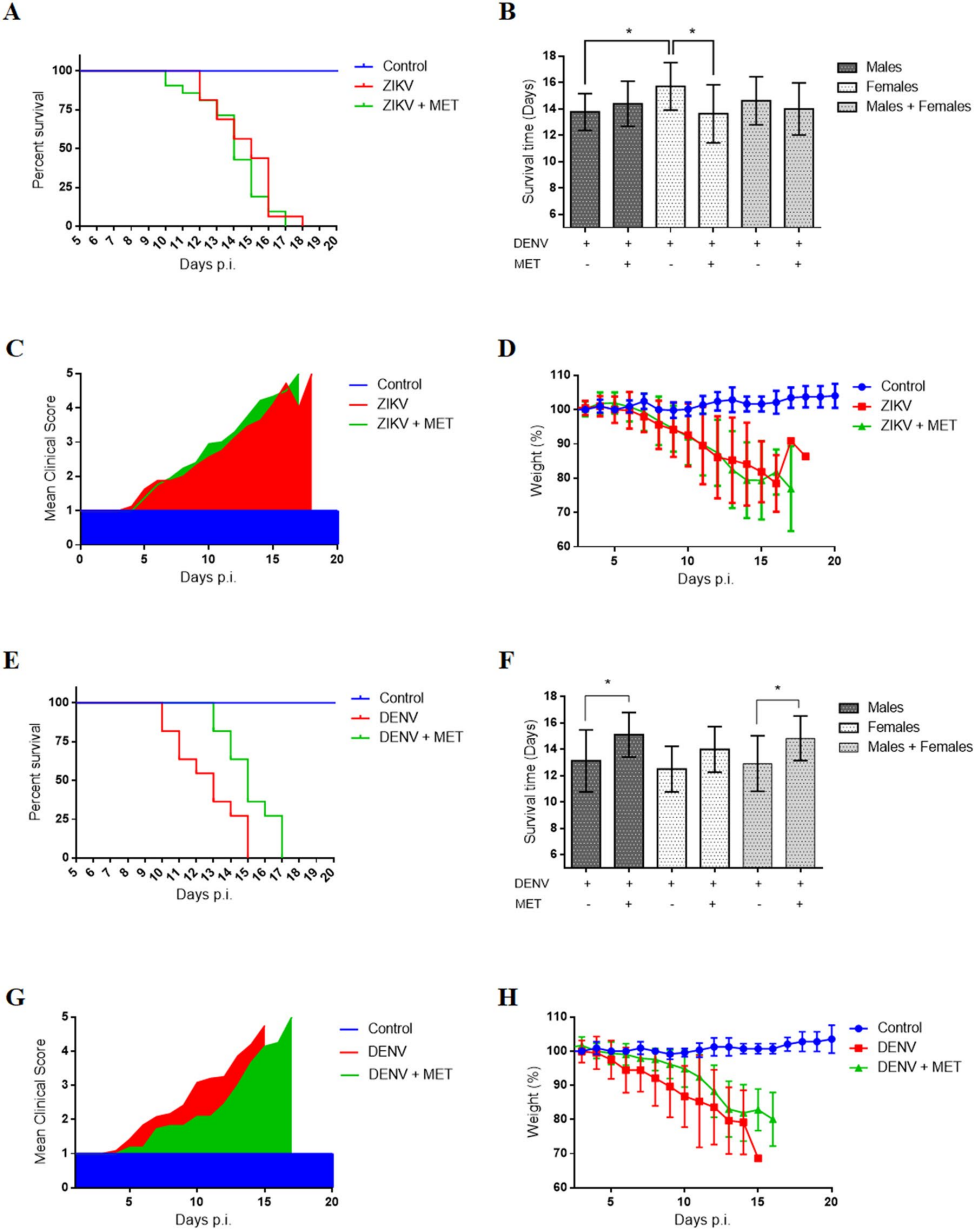
Effect of MET treatment on the survival of ZIKV- and DENV-infected AG129 mice. The “Control” group consisted of mice that were treated with MET and were not infected; the “ZIKV or DENV” group consisted of mice that were infected with each virus and untreated (H2O); the “ZIKV or DENV + MET” group consisted of mice that were inoculated with DENV or ZIKV and were treated with MET. (**A**) Kaplan–Meier curve, (**B**) average survival rate, (**C**) Mean clinical score, (**D**) and average body weight percentage of ZIKV-infected AG129 mice. (**E**) Kaplan–Meier curve, (**F**) average survival rate, (**G**) Mean clinical score, (**H**) and average body weight percentage of DENV-infected AG129 mice. The graphs represent groups of mice of both sexes, except graphs (**B**,**F**), which show the average survival rates of male, female, and both sexes, with and without MET treatment. Days post-infection (Days p.i.). * indicate statistically significant differences between the MET treated and untreated groups; the summary of survival trials is shown in Table 2.

The survival time of the ZIKV-infected mice showed no significant difference compared to the treated ones. The average survival rate, the signs of disease, and the weight loss were similar between treated and untreated mice (Fig. 6A–D), suggesting that, although MET inhibits ZIKV infection in cell culture, it failed to inhibit ZIKV infection in mice. Interestingly, we observed a shorter median survival (no significant) in ZIKV-infected and treated mice (14 days), compared to untreated ones (16 days), as well as a worsening of clinical signs (increased back and limb paralysis) in female mice (Table 2). For this reason, females and males were analyzed as separate groups. The summary of the results obtained from the survival trials by sexes is shown in Figure S2 and Table S1. This analysis allowed us to observe an increase in the severity of infection signs (Figure S2C or Table S1) and decreased average survival rate (Fig. 6B) only in ZIKV-infected and treated female mice compared to untreated females.

**Table 2:** Effect of MET treatment on ZIKV or DENV infected AG129 mice.

Treatment groups	# events (n)	Median survival (days)	Average survival rate (days)	Are the survival curves sig different?	Are the Average survival rate sig different?	Remarks
ZIKV	16 (9 ♂, 7 ♀)	16	14.63 ± 1.821	NO	NO	Slight exacerbation of clinical signs of ZIKV (increased paralysis of the back and extremities) in MET-treated female mice
ZIKV + MET	21 (10 ♂, 11 ♀)	14	14.00 ± 1.975			
DENV	12 (8 ♂, 3 ♀)	13	12.92 ± 2.10	YES Log-rank (Mantel-Cox) test * p = 0.0339 YES Gehan-Breslow-Wilcoxon test * p = 0.0358	YES ANOVA-LSD *p = 0.0197	Mice treated with MET showed mild signs of DENV and a higher median and median survival rate than untreated mice
DENV + MET	12 (9 ♂, 4 ♀)	15	14.83 ± 1.69			

Contrasting with ZIKV, MET treatment induced a clear improvement in DENV-infected mice (Fig. 6G), showing milder infection signs (little bristly hair, greater mobility, and absence of paralysis in lower limbs) compared to the untreated group. No significant weight-loss differences were detected between untreated and treated mice (Fig. 6H). However, the Log-rank and Wilcoxon test showed significant differences in the Kaplan–Meier survival curves (p = 0.0339 and p = 0.0358, respectively) between treated and untreated mice, with a median survival of 15 and 13 days p.i. respectively (Fig. 6E or Table 2). The average survival rate also increased significantly in treated mice (14.83 ± 1.69, p = 0.0197) compared to untreated mice (12.92 ± 2.10) (Fig. 6F or Table 2). The summary of the results obtained from the survival tests is shown in Table 2.

## Discussion

Diseases caused by flaviviruses remain one of the most important health problems worldwide. There is currently no vaccine or specific therapeutic agent against ZIKV, and the use of the DENV vaccine has been restricted only for seropositive individuals^6,7^. Therefore, the search for new strategies to control flavivirus infections is essential. In this sense, host-directed therapy is one of the tools used to inhibit viral replication.

Our group have demonstrated that MET, an FDA-approved drug used as an oral antidiabetic agent for treating type II diabetes, inhibits DENV in vitro infection^14^. In the present study, ZIKV infection was also inhibited by MET in Huh-7 and U-87 cells, being more effective in the neuronal lineage cells (IC_50_ = 5.84 mM) than in the liver cells (IC_50_ = 9.0) (Fig. 1); which could be advantageous since ZIKV is a neurotropic virus^25,26^. This finding was confirmed by the SI, based on the ratio CC_50_/IC_50_ (toxicity/potency of the drug), where the values for U-87 and Huh-7 cells were 4.68 and 3.65, respectively (Table 1).

The anti-ZIKV effect of MET on infected BeWo cells also suggested a cell-type-independent antiviral activity (Figure S1C). However, the infection efficacy (Figure S1B) and the drug's toxicity (Figure S1A) did not allow us to calculate MET SI in BeWo cells. This fact could be due to the different tolerance of the cell lines to the drug-induced reduction in cholesterol levels.

Regarding cellular toxicity, it is known that acute depletion of cholesterol synthesis by lipid-lowering drugs is usually toxic to cells since cholesterol is the main lipid constituent of the plasma membrane and is essential for cellular function. Therefore, we compared the effectiveness and toxicity of MET with LOV, another hypolipidemic drug candidate for treating DENV and ZIKV infections.

Overall, MET was less toxic than LOV. Even at low concentrations (1uM) LOV was highly toxic in U-87 and BeWo cells; thus, treatments in these cells were not possible. Interestingly, LOV treatment inhibited ZIKV infection in liver cells more effectively than MET treatment, obtaining a SI value of 11.15 (Figure S1D), threefold higher than the calculated value for MET (3.65) (Table 1). Two factors could explain this result: one is that since the mechanism of action of LOV is the specific inhibition of the HMGCR activity^27^, as mentioned above, the cholesterol levels in hepatic cells are directly affected by the drug; and the second is that the tolerance against cholesterol depletion of the cell lines is different, being Huh-7 cells highly tolerant because they are specialized in the synthesis and distribution of cholesterol^28^. Still, the low toxicity of MET and its antiviral property on other cell types indicate that it can be an excellent candidate for the treatment of ZIKV infection.

Concerning its antiviral property, MET did not reduce viral yield when it was pre-incubated with ZIKV neither when administered during viral adsorption, suggesting that this drug affects viral replication (figure S1E). In this sense, MET treatment significantly reduced the percentage of infected cells, viral yield, and viral protein synthesis in ZIKV-infected hepatic and neuronal cells at concentrations equal to or greater than 7.5 mM. (Fig. 2A–D). The statistical analysis between both infected cell lines correlated with the SI, showing greater MET effectiveness in U-87 cells than in Huh-7 (Fig. 2A,E).

Furthermore, our results demonstrated the capability of this drug to inhibit other flaviviruses (Fig. 3) by calculating IC_50_ (5.2 and 5.4 mM, respectively) and SI (6.32 and 6.09, respectively) for DENV and YFV (Fig. 3A,B or Table 1). The treatment also affected the percentage of infected cells and the viral yield of both viruses (Fig. 3C,D). It showed a higher antiviral efficacy against DENV than ZIKV (Fig. 3C,D,F). We cannot be sure that MET was more effective in inhibiting YFV infection compared to ZIKV because the infection efficacy of YFV was not the same as that of ZIKV or DENV (Figure S1B). In any case, its broad anti-flavivirus spectrum can be explained by the fact that MET inhibits the formation of replicative complexes of the three viruses (Figs. 4 and 5), as reported by Soto Acosta and collaborators on DENV^14^.

It has been reported that DENV replicative complexes formation requires cholesterol and other lipid species^11,12^; consequently, the drug treatments that reduce intracellular cholesterol decrease viral infection^13,14^. Likewise, the dependence on lipid synthesis has been confirmed for ZIKV^29–33^. Lipidomic analyses have shown alterations in the lipid profile in mosquito cells^29^ and fetal placental cells^33^ due to the reprogramming of lipidome during ZIKV infection, which provides fundamental building blocks for optimal biogenesis and function of the viral replicating organelles. Therefore, the down-regulation of lipid synthesis using hypolipidemic agents could reduce ZIKV infection^34^.

Here, it was observed that MET modified the distribution of viral proteins in the perinuclear region of the flavivirus-infected Huh-7 cells, causing a delocalization between the viral proteins in the replicative complex (Fig. 4A,B). The proximity between structural and nonstructural viral proteins in the endoplasmic reticulum membrane rearrangements ("replication complexes") is relevant for proper viral assembly^35^. The Pearson’s coefficient index indicated that there is colocalization between NS4A and E protein during ZIKV and YFV infection, supporting the replicative complexes' integrity before MET treatment^14^. This colocalization was lost during MET treatments, suggesting that this drug disrupts the integrity of the three viruses' replicative complexes. Moreover, ultrastructural analysis by TEM at 48 h revealed a decrease in the number of replicative complexes in infected cells treated with MET (Fig. 5A,B) compared to untreated cells. This reduction was similar in number to ZIKV-induced vesicles at early time points of infection (12 h) in Huh-7 cells^24^.

Finally, the effect of MET treatment on immunodeficient model mice was analyzed. MET treatment significantly increased (2 days) the average survival rate in DENV-infected and treated mice in contrast to untreated mice, with a median survival of 15 and 13 days, respectively (Fig. 6E,F or Table 2). Interestingly, MET treatment reduced the severe signs of DENV-infected mice, which presented mostly mild infection signs (little bristly hair, less lethargy, and absence of paralysis in lower limbs) (Fig. 6G). Supporting our results, a clinical study reported an association between the use of MET in diabetic patients and the lower risk of suffering a severe disease caused by DENV^22^. Together, these findings suggest that MET treatment could attenuate and/or prevent severe DENV infection forms.

Interestingly, MET treatment failed to prevent or delay ZIKV-infected mice mortality and did not counteract any disease signs (Fig. 6A–D). One possible explanation is that MET acts primarily in the liver, one of the main target organs of DENV but not of ZIKV infection^18,19,36^. Contrary to expectations, we observed signs of severity and decreased survival rate only in treated ZIKV-infected females compared to untreated females (Fig. 6B and Table S1). The explanation for this specific fact could be related to two factors: (1) The differential immune response between females and males, and (2) The contrasting regulation of AMPK in different organs by the viruses.

The first is based on a disparate immune response between sexes to certain infections^37,38^. For instance, female sex has been associated with severe DENV^39^ and a higher frequency of symptomatic ZIKV infections^40^. In this context, studies with mice have suggested that females are mostly affected by ZIKV^41^. Snyder-Keller et al. (2019) reported increased cell death in female 129S1 mice brains compared to male mice. Although there was no evidence that ZIKV levels were higher in female than in male mice, the neuropathological events that triggered more significant necrosis and accumulation of calcifications in the brains of female mice were notable^41^.

The second is based on the AMPK protein, which may be up-or down-regulated in various infections. It has been reported that hepatitis C virus (HCV)^42,43^, Epstein-Bar virus (EBV)^44^, and DENV^14^ can downregulate the active form of AMPK, phosphorylated at Thr-172 (pAMPK). Such inactivation provides a favorable host lipid environment for replication. On the contrary, human cytomegalovirus (HCMV) infection increases AMPK activation for glucose import and the glycolytic pathway, essential during HCMV replication^45^.

Interestingly, different effects on AMPK protein during ZIKV infection have been reported. Cheng et al. (2018) reported that during ZIKV infection, there is a decrease of pAMPK in HRvEC and HUVEC cells. In this regard, pharmacological activation of AMPK using GSK621, AICAR, or MET induces the reduction of ZIKV^46,47^ and DENV^12^ replication in HUVEC and Huh-7 cells, respectively. However, Thaker et al. have reported an increase in the active form of pAMPK during ZIKV infection in brain tissue of Infar1 -/- mice, in human HFF-1 (foreskin fibroblast cell line) and hFRPE cells (human fetal retinal pigment epithelial). The latter study suggested that the activation of AMPK promotes caspase-mediated cell death^48^.

It remains to be determined whether ZIKV can differentially modulate AMPK protein in different organs. In this case, MET could have a dual effect, counteracting infection in specific tissues and contributing to pathology and cell death in others. Considering all the preceding, the worsening of neurological signs of ZIKV in female mice will require further studies.

Finally, it is relevant to mention a new antiviral property that has been attributed to MET. Evidence shows that interferon actively represses sterol biosynthesis as an antiviral strategy^49^. Conversely, limiting flux through the cholesterol biosynthetic pathway spontaneously engages a type I IFN response, creating a metabolic-inflammatory circuit that links perturbations in cholesterol biosynthesis with the activation of innate immunity^50^, in which the AMPK protein plays a regulatory role^51^. In this regard, besides restricting the lipid^14^ and energy^52^ resources necessary for viral replication, MET could potentiate the innate immune response through AMPK activation^52,53^. In this case, AG129 mice would not be the most appropriate model to determine Metformin's antiviral efficiency against ZIKV. However, the present study demonstrated that MET reduces the negative signs of DENV disease and increases the survival time of AG129 deficient mice, suggesting that at least one of its anti-DENV mechanism is independent of the IFNs-mediated immune response. In contrast, further study is needed to elucidate the mechanisms underlying this drug during ZIKV infections.

## Conclusion

Here, we report the in vitro and in vivo antiviral potential of MET against ZIKV compared to the effect against DENV. The in vitro results showed that MET inhibits ZIKV infection in three cell lines, suggesting that this effect persists regardless of cell type. At the same time, its effectiveness depends on its toxicity, most likely related to each cell's tolerance to cholesterol depletion. We also demonstrate the broad anti-flavivirus spectrum of MET and its greater effectiveness in inhibiting DENV in the in vitro and in vivo model. Interestingly, MET did not counteract the negative signs of ZIKV disease, but it increased the survival time of DENV-infected mice and decreased the severity of DENV disease. Together, these findings indicate that MET is an effective antiviral agent to inhibit DENV infection. Here we remark that although DENV and ZIKV belong to the same family, one drug can cause different effects on each virus. Therefore, it is necessary to continue studying the molecular mechanisms that the drugs exert on each virus to know each one's effectiveness and risk during flavivirus infections.

## Material and methods

### Cell culture, viral strains, and drugs

#### Cell culture

The Huh-7 cell line (Human hepatoma-derived cells, Kindly donated by Dr. Ana Maria Rivas, Universidad Autónoma de Nuevo León) and the U-87 cell line (Glioblastoma cells, Kindly donated by Dr. Olivia Hernández-González, Instituto Nacional de Rehabilitación, Mexico) were grown in Advanced Dulbecco’s modified Eagle´s medium (DMEM, Gibco) supplemented with 2 mM glutamine, penicillin (5 × 10^4^ U/mL, Sigma), streptomycin (50 μg/mL, Sigma), 10% fetal bovine serum (FBS, Gibco) and 1 mL/L of amphotericin B (Fungizone, Gibco) at 37 °C and a 5% CO_2_ atmosphere. The BeWo Cells (Human Choriocarcinoma, kindly donated by Dr Federico Martínez Montes, Universidad Nacional Autónoma de México) were cultivated with medium Kaighn´s Modification of Ham´s F12 (F-12K, ATCC) supplemented and incubated under the same conditions. All cell lines were cultured under the same conditions in 24- or 12-well plates for the trials.

#### Virus

The ZIKV (MEX_CIENI551, kindly provided by Dr. Jesús Torres, Escuela Nacional de Ciencias Biológicas del Instituto Politécnico Nacional) and the DENV (serotype 2 New Guinea strain) propagation were carried out in CD1 suckling mice brains (provided by Unidad de Producción y Experimentación de Animales de Laboratorio (UPEAL)). The YFV 17D (live-attenuated vaccine) propagation was carried out in C6/36 Aedes albopictus cells adapted to grow at 35 °C^54^. The DENV and ZIKV titers were determined by foci forming units (FFU) assay in Huh-7 cells^55^, while YFV titers by plaque-forming units (PFU) assays^56^. The CD1 suckling mice brains from mock-infected mice or complete medium for C6/36 cells were used as control.

#### Drugs

MET and LOV were obtained from Abcam Biochemicals (catalogue number ab120847 and ab120614, respectively). MET was dissolved in water to prepare a stock solution at 100 mM. LOV was dissolved in DMSO to prepare a stock solution at 50 μM.

### Flavivirus infection and treatment

The cell lines were seeded in 12-wells (5.68 × 10^5^ Cells) or 24-wells (2.72X10^4^ Cells) plate format at 70–80% of confluence. They were grown for 24–36 h and then infected with either ZIKV or YFV, or DENV at a multiplicity of infection (MOI) of 5 in medium supplemented with 1% of FBS for two h at 37 °C. Later, the cells were washed three times with HANKS (Gibco). For the treatments, the drug was diluted in complete medium to obtain the desired concentration, and the cells were incubated with the drugs for 24 or 48 h at 37 °C. Sterile water (MET) or DMSO (LOV) was used as a vehicle in infection controls. Three independent experiments in duplicate were performed for each assay.

### Flow cytometry and confocal microscopy

The infected untreated and treated cells were analyzed by flow cytometry and confocal microscopy to determine the percentage of infected cells and viral protein localization. The samples were processed as previously reported by Soto-Acosta et al.^14^. Mouse anti-Env 4G2 monoclonal antibody (MAB10216) was used to determine the percentage of infected cells by flow cytometry. The 4G2 antibody was also used with rabbit polyclonal antiNS4A antibody (GeneTex) to determine viral proteins' colocalization in replication complexes by confocal microscopy. As secondary antibodies, a goat anti-mouse Alexa Fluor 488, a goat anti-mouse Alexa Flour 405 and a goat anti-rabbit Alexa Flour 555 were used (Life Technologies). The nuclei were counterstained with Hoechst 33342 (Life Technologies). The Flow cytometry was performed in a BD LSR Fortessa, and the data were analyzed using the FlowJo v. 10 software. The infected cells were observed in a Zeiss LSM700 laser confocal microscopy to determine colocalization ratios between viral proteins, and images were analyzed with ZEN software v. 2010. Three independent experiments in duplicate were performed to determine the percentage of infected cells, and two independent experiments duplicated for the viral protein localization.

### Cytotoxicity (CC_50_) and inhibition concentration (IC_50_)

The 50% cytotoxic concentration (CC_50_) in cells treated with vehicle or with increasing concentrations of MET (0, 1, 2.5, 5, 7.5, 10, 12. 5, 15, 20, and 25 mM) was calculated at 24 h by a colorimetric assay (Roche 11 465 007 001) based on the MTT method ((3-(4,5-dimethylthiazol-2-yl)-2,5-diphenyltetrazolium bromide), according to the manufacturer's instructions using spectrophotometry (BioTek ELx800) measuring absorbance at 540 nm. The Half-maximal (50%) inhibitory concentration (IC_50_) was calculated using the same concentrations in ZIKV or DENV infected cells. Subsequently, the cells were analyzed by flow cytometry. The CC_50_ and IC_50_ of LOV was calculated using the following concentrations: CC_50_ = 15, 25, 35, 75, 100, 150, 200 and 400 μM; IC_50_ = 1, 6, 12, 18, 25, 35, 50, 75, 100, 150, 200 μM. The CC_50_ and IC_50_ were estimated by linear regression analysis. Three independent experiments in duplicate were performed. The Data were analyzed and plotted with Graph Pad Prism software version 6.0.

### Viral yield

Supernatants from the DENV and the ZIKV infected untreated, and treated cells were used to determine the viral yield using foci forming units (FFU) assay, following the methodology previously reported by De Jesús-González et al.^55^. Supernatants from YFV-infected untreated and treated cells were used to determine the viral yield using plaque-forming units (PFU) modified assay of Morens et al.^56^. Three independent experiments in duplicate were performed for each assay.

### Western blot assay

The cells were infected with ZIKV or DENV at a MOI of 5 and treated with MET (0, 1, 7.5, and 15 mM) for 24 h. Subsequently, cell lysates were obtained with RIPA buffer and 35 μg of protein extract were used for Western blot assays following the methodology previously reported by De Jesús-González et al.^55^. The Rabbit anti-NS3 polyclonal antibody (GTX124252) was used to detect ZIKV and DENV infection, and the anti-rabbit HRP (Cell Signaling) was used as a secondary antibody. The Rabbit anti-GAPDH and anti-Tubulin antibodies (Cell Signaling) were used as a loading control. The proteins were detected using Super Signal West Femto Chemiluminescent Substrate (Thermo Scientific). Three independent experiments in duplicate were performed. The densitometric analysis was performed using the myImageAnalysis software (Thermo Fisher Scientific, Illinois, USA), and the results were plotted with Graph Pad Prism software version 6.0.

### Transmission electron microscopy

Mock-infected, infected untreated, and MET-treated Huh-7 cells were incubated for 48 h and processed using the methodology previously reported by Reyes-Ruiz et al.^57^. Thin sections (70 nm) were stained with uranyl acetate and lead citrate and visualized in a Jeol JEM-1011 transmission electron microscope. Two independent experiments in duplicate were performed.

### AG129 mouse survival assays

The AG129 mice from 6 to 8 weeks of age were kindly donated by Dr. Marco Antonio Meraz Ríos (Biomedicine Department, Center for Research and Advanced Studies (CINVESTAV-IPN-México), and provided by the Laboratory Animal Production and Experimentation Unit (UPEAL). The AG129 mice were infected with 2 × 10^7^ FFU of ZIKV or 4 × 10^6^ FFU of DENV per mouse. The viruses were inoculated intraperitoneally in a volume of 100 μL of injectable water. The MET-treated group received 50 mg/kg/day of the drug, according to literature^58^, using an oral tube. The treatments started from day four post-infection for ten days. The mice's weight and the signs of the disease were monitored daily until the day of euthanasia. The signs of the disease are based on a clinical score reported by Orozco et al.^59^, to monitor the average morbidity of DENV-infected mice on a scale of 1 to 5, where “1” represents healthy and “5” moribund mice, so it was modified and adapted for ZIKV-infected AG129 mice (Table S2). Three independent essays of 4 mice each were performed, without gender equity. Two independent assays by age groups, 6-week-old mice and 8-week-old mice, were performed for ZIKV. There were no differences between both groups; therefore, the data were analyzed in the age range of 6 to 8 weeks. Mice that died from causes other than infection were excluded. The survival results, the clinical score, and the mouse weight were plotted and analyzed using the Graph Pad Prism software version 6.0.

### Statistical analysis

For the statistical analysis, numerical data were expressed with means and standard deviations (SD). The results were normalized and presented in percentages considering 100% of the infected cells untreated. To compare treated and untreated cells, an analysis of variance (ANOVA) was performed using the Dunnett's multiple comparisons tests; to compare treatments between both cell types, an analysis of variance (ANOVA) was performed using Sidak's multiple comparisons tests. The student t-test was used to compare the treated and untreated groups in confocal and electron microscopy. For the in vivo assays, the Kaplan–Meier survival curves were drawn, and the Wilcoxon test and Mantel-Cox test were used to compare the survival between treated and untreated groups. Finally, to compare the average survival rates between treated and untreated groups, the ANOVA-LSD test was used. In all cases, a p ≤ 0.05 was considered statistically significant.

### Ethics statement

This study was conducted following the Official Mexican Standard Guidelines for Production, Care and Use of Laboratory Animals (NOM-062-ZOO-1999). The protocol number 048-02 and 0305-19 were reviewed and approved by the institutional committee for the care and use of animals (CINVESTAV IACUC/ethics committee CINVESTAV-IPN, Mexico).

All methods are reported in accordance with **ARRIVE** guidelines (https://arriveguidelines.org).

## Supplementary Information

Supplementary Information
