# Supplementary material for: The antiviral effect of metformin on zika and dengue virus infection
Source: Sci Rep. 2021 Apr 22;11:8743. doi: 10.1038/s41598-021-87707-9 (PMC8062493; doi:10.1038/s41598-021-87707-9)
Supplement: Supplementary file 1 — Supplementary Information [file 41598_2021_87707_MOESM1_ESM.docx]

**The antiviral Effect of Metformin on Zika and Dengue virus**

Carlos Noe Farfan-Morales,^1^ Carlos Daniel Cordero-Rivera,^1^ Juan Fidel Osuna-Ramos,^1^ Irma Eloisa Monroy-Muñoz,^2^ Luis Adrián De Jesús-González,^1^ José Esteban Muñoz-Medina,^3^ Arianna M. Hurtado-Monzón, ^1^ José Manuel Reyes-Ruiz,^1^ and Rosa María del Ángel^1^ *

1. Department of Infectomics and Molecular Pathogenesis, Center for Research and Advanced Studies (CINVESTAV-IPN), Mexico City.
2. Department of Genetics and Human Genomics, National Institute of Perinatology "Isidro Espinosa de los Reyes." Mexico City.
3. Central Laboratory of Epidemiology, National Medical Center La Raza, Mexican Institute of Social Security, Mexico City.

*Address correspondence to Rosa María del Ángel ([rmangel@cinvestav.mx](mailto:rmangel@cinvestav.mx)).

Supplemental Material


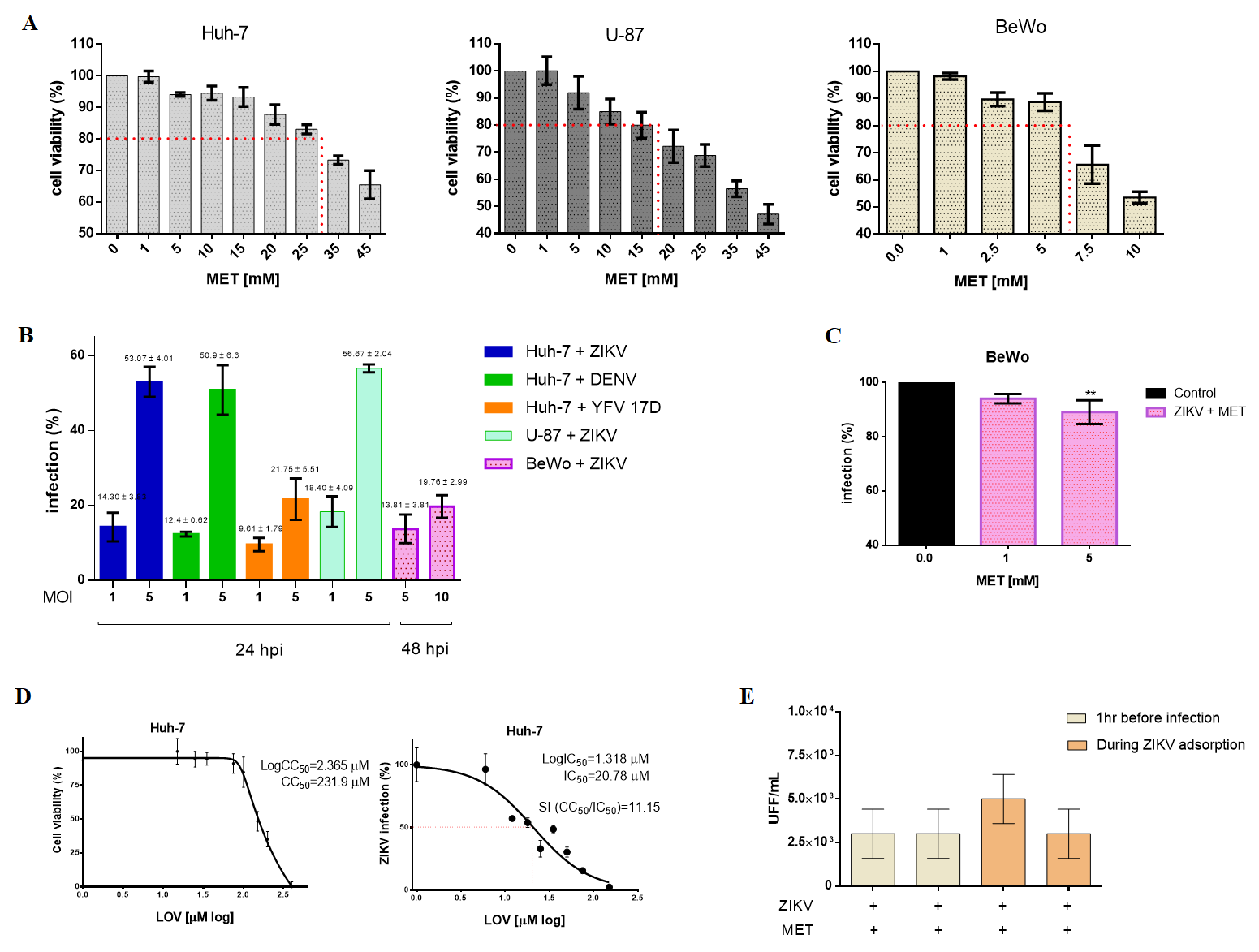


**Figure S1.** (A) Cell viability in Huh-7, U-87 and BeWo cells. (B) The efficiency of *in vitro* infection of the ZIKV, DENV, and YFV viruses. (C) MET treatment in BeWo cells infected with ZIKV. (D) The CC_50_ and IC_50_ of lovastatin in ZIKV-infected Huh-7 cells. (E) Metfomine incubation (9 mM) with ZIKV, one hour before adsorption and during viral adsorption (2 hrs).

**
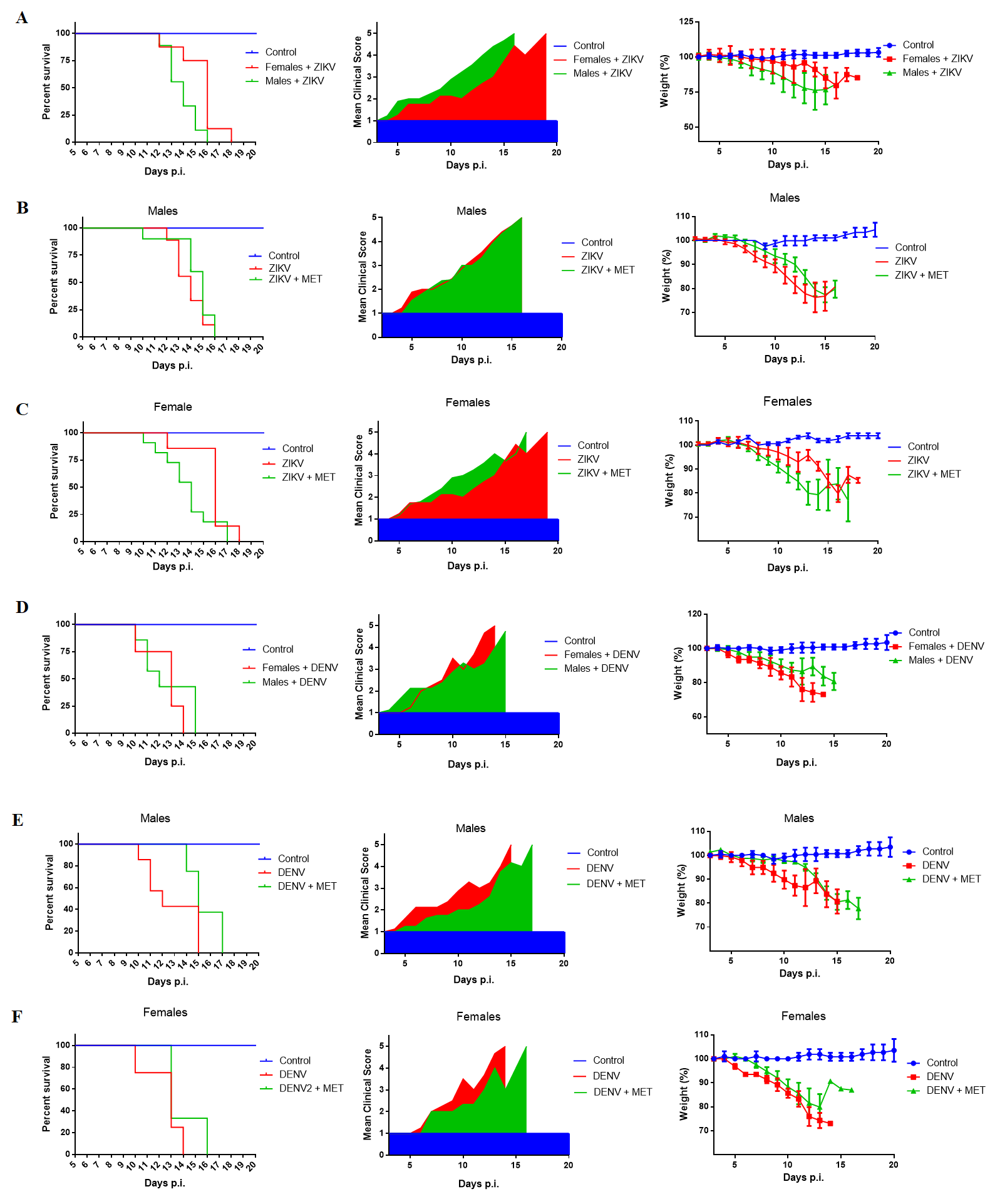
**

**Figure S2.** MET treatment on male and female AG129 mice infected with ZIKV or DENV. The “Control” group consisted of mice that were treated with MET and were not infected; the “ZIKV or DENV” group consisted of mice (Males or Females) that were infected with each virus and untreated (H2O); the “ZIKV or DENV + MET” group consisted of mice (Males or Females) that were inoculated with DENV or ZIKV and were treated with MET. The figure shows the Kaplan-Meier curve, Mean clinical score, and average body weight percentage of (A) male and female mice infected with ZIKV; (B) MET treatment in ZIKA-infected male mice; (C) MET treatment in ZIKA-infected female mice; (D) Male and female mice infected with DENV; (E) MET treatment in DENV-infected male mice; (F) MET treatment in DENV-infected female mice. Days post-infection (Days p.i.). The summary of survival trials is shown in Table S1.


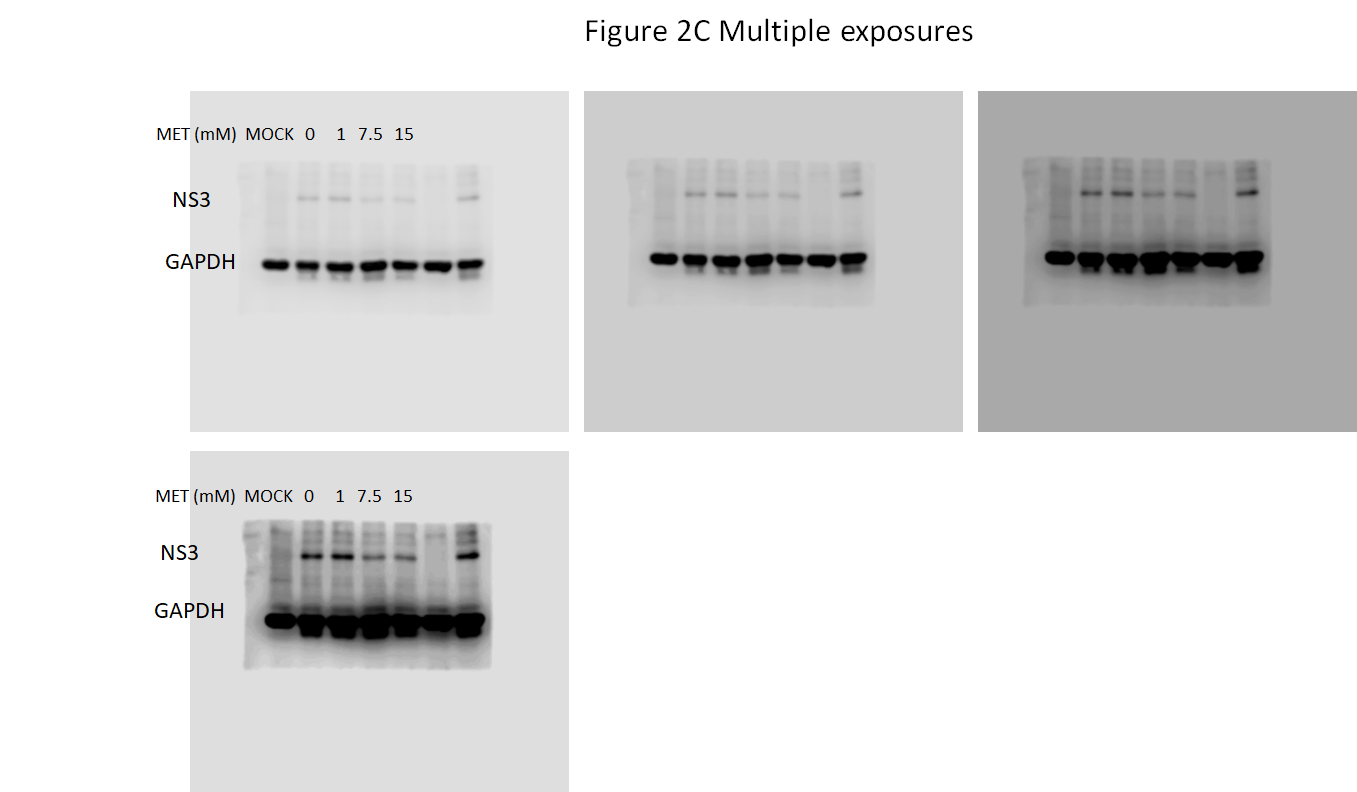


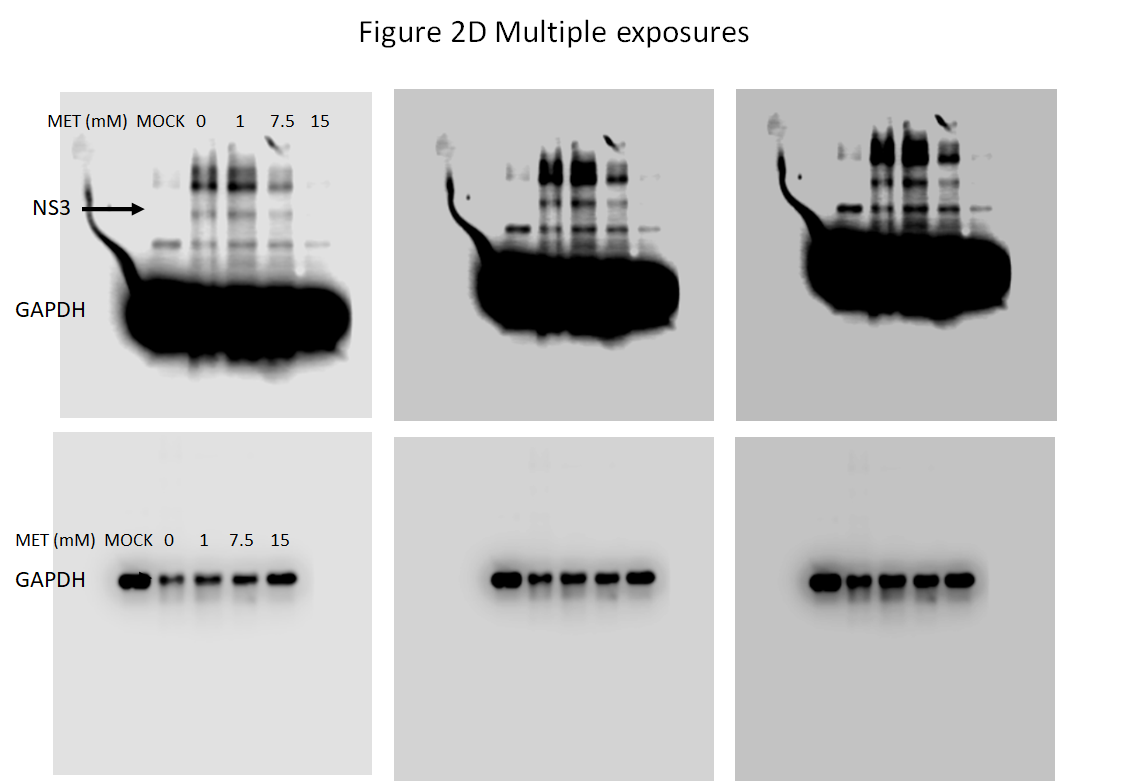


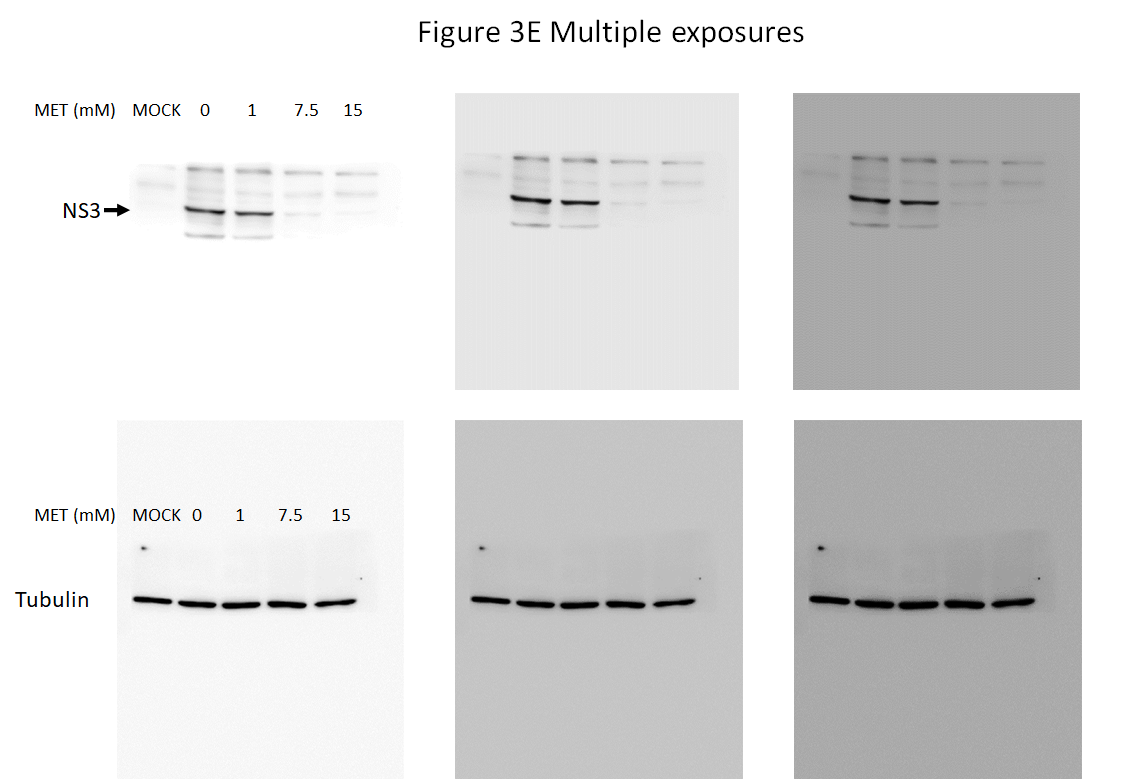


| Table S1. MET treatment on male and female AG129 mice infected with ZIKV or DENV. | | | | | | | |  |
| --- | --- | --- | --- | --- | --- | --- | --- | --- |
| *Mice* | | *Treatment Groups* | *# events (n)* | *Median survival* | *Average survival rate* | *Are the survival*  *curves sig different?* | *Are the Average survival rate sig different?* | *Remarks* |
| Male | ZIKV | | 9 | 14 | 13.78±1.394 | NO | NO | There were no changes between groups |
|  | ZIKV + MET | | 10 | 15 | 14.40±1.713 |  |  |  |
| Female | ZIKV | | 7 | 16 | 15.71±1.799 | NO | YES ANOVA-LSD *p=0.0246 | MET-treated mice showed severe signs of ZIKV infection compared to untreated |
|  | ZIKV + MET | | 11 | 14 | 13.64±2.203 |  |  |  |
| Male | DENV | | 8 | 13.5 | 13.13±2.35 | NO | YES ANOVA-LSD *p=0.0407 | MET-treated mice showed a milder disease of DENV compared to the untreated. |
|  | DENV + MET | | 9 | 15 | 15.11±1.69 |  |  |  |
| Female | DENV | | 4 | 13 | 12.50±1.73 | NO | NO | MET-treated mice showed a milder disease of DENV compared to the untreated. |
|  | DENV + MET | | 3 | 13 | 14±1.73 |  |  |  |

| **Table S2. Morbidity scale in AG129 mice** | | |
| --- | --- | --- |
| Score | Clinical signs of DENV disease | Clinical signs of ZIKV disease |
| 1 | healthy | healthy |
| 2 | Mild signs of lethargy | bristly hair |
| 3 | Lethargy, bristly hair, and stooped posture | bristly hair, slight signs of lethargy at rest with stimulus-induced hyperactivity (signs of stress) |
| 4 | lethargy, bristly hair, stooped posture, muscle weakness and reduced mobility, poor response to stimuli | bristly hair, lethargy, stooped posture, muscle weakness and reduced mobility at rest. They still have mobility and response to stimuli. |
| 5 | Moribund: Lethargic, very bristly hair, not able to stand up, no mobility, and no response to stimuli. | Moribund: Lethargic, very bristly hair, not able to stand up, with or without mobility in the face of stimuli |
| Exacerbated signs may appear during the disease and are characterized as neurological signs:  *Paralysis of a limb  ** paralysis of both or more limbs  *** Paralysis of one or more limbs with tremors or ataxia. | | |
